# A Comprehensive Protocol for Improving the Description of Saprolegniales (Oomycota): Two Practical Examples (*Saprolegnia aenigmatica* sp. nov. and *Saprolegnia racemosa* sp. nov.)

**DOI:** 10.1371/journal.pone.0132999

**Published:** 2015-07-17

**Authors:** Jose Vladimir Sandoval-Sierra, Javier Diéguez-Uribeondo

**Affiliations:** Departamento de Micología, Real Jardín Botánico-CSIC, Madrid, Spain; Uppsala University, SWEDEN

## Abstract

The description, identification and classification of organisms are the pillar in biodiversity and evolutionary studies. The fungal-like organism *Saprolegnia* contains important animal pathogens. However, its taxonomy is weak, making it difficult to perform further studies. This problem mainly arises from the unavailability of suitable holotypes. We propose a standardized protocol for describing *Saprolegnia* spp. that includes good cultural practices and proper holotype preservation. In order to illustrate this new proposal, we describe two species, *Saprolegnia aenigmatica* sp. nov. and *Saprolegnia racemosa* sp. nov., based on the recently described molecular operational taxonomic units (MOTUs), phylogenetic relationships, and the analyses of morphological features. We show that they belong to two different MOTUs that are grouped into two sister clades. Morphologically, we find that *S*. *racemosa* exhibits a species-specific character, *i*.*e*., aggrupation of oogonia in racemes, while *S*. *aenigmatica* does not have any specific characters. Analyses of a combined set of characters, *i*.*e*., length and breadth of sporangia, length/breadth ratio (l/b) of oogonia, cyst and oospore diameter, and the number of oospores per oogomium, allow distinguishing these two species. To improve *Saprolegnia* taxonomy, we propose to incorporate into the protologue: (i) several isolates of the new species; (ii) the rDNA sequences to compare them to data-bases of *Saprolegnia* sequences of reference; (iii) a phylogenetic analysis to check relationships with other species; (iv) to preserve holotypes in absolute ethanol and to include lyophilized material from holotype; and (v) the *ex-type* as a pure culture from single-spore isolates stored in at least two different collections.

## Introduction

The genus *Saprolegnia* (Saprolegniales: Oomycota) comprises 23–24 species [1, 2], which seem to be widely distributed throughout the world [2]. This genus contains important pathogens that are reportedly responsible for high-profiled declines in animal wildlife and aquaculture populations [3, 4]. In particular, the genus *Saprolegnia* possesses pathogenic species that attack the embryonic and adult stages of most fish worldwide, and as result of climate change are involved in the decline of amphibian populations [5–8]. In spite of the increasing need for a better understanding of the biology of these organisms, they lack of a robust taxonomy. This represents the main problem for studying them and performing deeper and detailed molecular and genomic analyses. The two main reasons for the lack of a robust taxonomy are:

1. The main features used for defining species (*e*.*g*., the morphological characters of sexual reproductive structures such as oogonium ornamentation, lipid droplets position in the oospore and antheridial branch origin) [1, 2, 9], are often absent, ambiguous, highly variable, and overlap among species [10, 11]. This has resulted in a lack of consensus among researchers giving raise to a great number of misidentified specimens [10, 12]. Moreover, the majority of types in *Saprolegnia* consist of illustrations or metabolically inactive material, which are according to the International Code of Nomenclature [13]. That way of type preserved does not always maintain the features attributed to the taxon. These features could be reproduced if protologues also included pure cultures of the type as *ex-types*. However, only some of the species described after 1977 have included such cultures. Therefore, existing holotype descriptions do not always allow assigning an isolate to an existing species, or to a new species of *Saprolegnia*.
2. As is the case for many microorganisms, information regarding the DNA sequences of the holotypes has not always been included in the protologues. In *Saprolegnia*, the majority of holotypes are not suitable for DNA extraction, and protologues either do not include cultures as *ex-types*, or these are not easily available. Obtaining DNA sequence information from the type specimens is of crucial importance. Recent molecular analyses of a large number of isolates have resolved some of the taxonomic problems of this group [10, 12, 14], and answered some key questions by defining molecular operational taxonomic units, MOTU based on ITS rDNA [12]. These studies pointed out that some species names could be synonyms of already described species [10, 12].

These two problems show that building a robust taxonomy in the *Saprolegnia* genus requires a standardization of the methodology to preserve type material. Therefore, the preserve types should include specimens with well-preserved specific morphological characters. Also, the protologues should enclose suitable source for DNA and the possibility to re-investigate a living culture. These requirements may be fulfilled by, for example: (i) preservation in absolute ethanol to maintain the morphological features, (ii) applying an additional method allowing molecular analyses, *e*.*g*., lyophilization of additional material from type, and (iii) including cultures as *ex-types* allowing repeatability of morphological structures and molecular analyses.

A number of good cultural practices as standards in taxonomic studies of organisms requiring morphological and molecular data are recommended [15]. These consist of: (i) including *ex-type* cultures of the species, wherever available, (ii) including *ex-types* such as single-spore isolates in order to avoid the presence of other similar species in the studied sample, (iii) deposit *ex-type* strains in at least one major international service culture collection, and (iv) provide sequence data of the holotype and the resulting sequence alignments and deposit them in a major international databases, such as GenBank [16], making them available to other researchers.

The aim of this work is to propose a standardized approach to describe *Saprolegnia* species by including good cultural practices and proper holotypes in the protologues. For this purpose, we provide two practical examples of the description of two new species corresponding to the MOTUs formerly designated as *Saprolegnia* sp. 2 and *Saprolegnia* sp. 3 [12], which might be involved in pathogenicity of aquatic animals, using both morphological and molecular characters.

## Material and Methods

### Sampling and isolation

This study includes 104 isolates of two undescribed species, corresponding to the MOTUs designated as *Saprolegnia* sp. 2 and *Saprolegnia* sp. 3 [12]. The specimens of these two new species are deposited in the culture collection of the Real Jardín Botánico RJB-CSIC of Madrid, Spain. The isolates were collected from diverse geographical freshwater aquatic habitats and hosts (S1 Table). All isolates were obtained as described in [5, 6, 12]. Briefly, in freshwater aquatic habitats, sampling was carried out by taking 300 mL of water using plastic bottles of 500 mL that contained rice seeds as bait. Rice seeds were incubated in the bottle at 20°C for 3 d until a superficial “cotton like” growth was observed. Then, the colonized rice seeds with a “cotton like” growth were place onto a peptone glucose agar plate (PGA) supplemented with antibiotics [5, 6]. For isolation from diseased eggs and tissues of amphibian and fish, these were first washed with sterile distilled water supplemented with 100 mg/L penicillin C and placed onto PGA plates as described above. From the resulting growing mycelia a selected agar plug (0,5 mm) was cut out and, then, transferred onto a new PGA plate [5, 6]. This procedure was repeated until no bacterial growth was observed in the plate.

### Preservation of morphological and molecular characters

#### Single-spore isolation

For a suitable preservation of morphological and molecular characters, selected isolates were first subjected to single-spore isolations. Colonies were grown in 0.5 mL droplets of peptone and glucose liquid media (PG-1) for 24 h at 15°C. Sporulation was induced by washing the mycelia with autoclaved river water three times, and then incubated in autoclaved river water for 15 h at 15°C to allow release of zoospores. In order to obtain single-spore isolates, a volume of 10–20 μL of zoospore suspension was transferred onto peptone, glucose and agar (PGA) plate, and spread using a glass plate spreader. The plate surface was examined at 100x using an inverted microscope (Zeiss Axiovert 25, Carl Zeiss, Germany), and a piece of agar of about 2.5 mm x 2.5 mm containing one individual germinating cyst was excised with a glass capilar. The excised piece was placed into 9 mm Petri dishes containing 10 mL of PG-1, and incubated at room temperature for 48 h. All pure cultures from a single-spore isolate were used as *ex-types* for a species description, and deposited in the culture collection at the Real Jardín Botánico, Madrid, Spain (S1 Table).

#### Morphological characters

Morphological characters (*e*.*g*. oogonia, antheridia, sporangia and gemmae) were obtained from selected single-spore isolates representing the different ribotypes (see below). Potential new species isolates were allowed to grow into new PGA plates with autoclaved hemp seeds on it, at room temperature, for 10–15 d. When the growing hyphae colonized the hemp seeds, these were transferred into 9 mm Petri dishes containing 10 mL of autoclaved river water. Each Petri dish was then incubated at selected temperatures of 5, 10, 15, 20 and 25°C, and checked every day on an inverted microscope for oogonia production.

#### Preservation methods

The type specimens were stored as required by the International Code of Nomenclature [13] in a metabolically inactive state. For this aim the morphological features were preserved in absolute ethanol. First, mycelia with characteristic features were fixed in 2% glutaraldehyde for 20 min, washed in distilled sterile water, and then dehydrated in a series of ethanol (30, 50, 70, 80, 90, 95 and 100%) solutions. The isolates were preserved in 100% ethanol in sterile 2.0 mL tubes with screw caps containing an o-ring seal. The preservation of morphological characters in one isolate was examined after 12 months. Data of preserved material such as oogonial length and breadth, oospore diameter, number of oospores per oogonium, antheridia and oospore types, were compared with those features on fresh material. For this comparison a one-way ANOVA analysis was implemented. The analysis was performed in R v 3.02 [17], using the MASS package v 7.3–37 [18]. Additionally, lyophilized material from type specimens was included to allow preserving the DNA. This was done by excising a piece of agar with growing hyphae with characteristic features and transferring it into a new plate of 10 mL of PG-l and incubating for 3 d at 15°C. The resulting mycelia were lyophilized for 2 d in 2.0 mL microtubes using a lyophilizer (VirTis BenchTop, Germany).

### Characterization of morphological features

Isolates with morphological structures of taxonomic value were mounted on glass slides, covered with a cover slip, and observed using a microscope (Olympus BX51, Olympus Optical, Tokyo, Japan) with transmitted bright-field illumination at magnifications of 200x or 400x. All characters were photographed using a Micropublisher 5.0 (Qimaging, Burnaby, BC, Canada) digital camera, and measurements were made applying the software ImageJ 1.48v [19]. For each isolate, 30 measurements were taken of the following characters: length and breadth of sporangia, length/breadth ratio (l/b) of oogonia, cyst and oospore diameter, and the number of oospores per oogonium. Nomenclature and descriptions linked to taxonomic variations were deposited in MycoBank [20].

A linear model analysis was used to evaluate interspecies differences: first, to determine variations of each individual character, and secondly, asses variations between the whole set of characters. A linear discriminant analysis was implemented to assess the probability of each value of a character to belong to particular species. The analyses were performed in R v 3.02 [17], linear model and linear discriminant analyses were performed using the MASS package v 7.3–37 [18], and the linear discriminant analysis graph was plotted using the ggplot2 package v 1.0.0 [21].

For detailed morphological descriptions, samples containing structures of interest (*i*.*e*., oogonia and antheridia) were visualized using a scanning electron microscopy (SEM) Hitachi s3000N (Real Jardín Botánico, CSIC, Madrid, Spain). Isolates preserved in absolute ethanol were critical-point dried and the material was sputter coated in a vacuum with an electrically conductive layer of gold to a thickness of about 80 nm. Samples were observed at a beam specimen angle of 45° with an accelerating voltage of 20kV and final aperture at 200 μm.

### Molecular and phylogenetic analyses

DNA extractions were carried out from the lyophilized material obtained as described above. The ITS rDNA was amplified using universal primers for eukaryotes ITS5 and ITS4 [22] under the conditions described in [12]. Phylogenetic analyses included 104 sequences from selected isolates (S1 Table), 31 GenBank sequences corresponding to MOTUs *Saprolegnia* sp. 2 and *Saprolegnia* sp. 3 [12] (Table 1), and 20 reference sequences for other MOTUs of *Saprolegnia* spp. [12]. The consensus sequences for ITS rDNA region were assembled and edited using Geneious v6.14 [23]. Phylogenetic analysis of sequences was performed using Bayesian inference and Maximum Likelihood analyses. For Bayesian inference analysis, the nucleotide model evolution GTR+Gamma was obtained by running the data sets in jModelTest 2 [24]. Bayesian inference analysis was carried out with MrBayes v3.2.1 [25]. The Bayesian inference analysis was implemented from three running with 10 million generation. For each run eight Markov chain Monte Carlo (MCMC) were done. The partitioned datasets were sampled every 1000th generation. The burn-in threshold was assessed analyzing the parameter files in the program Tracer v1.5.0 (http://tree.bio.ed.ac.uk/software/tracer/). The first 25% of the trees was discarded for each run, while the remaining trees were used to estimate branch lengths and posterior probabilities (pp). The Maximum Likelihood analysis was carried out with RaxML [26], with a random starting tree, employing the GTR+Gamma option, and clade support was assessed with 1000 bootstrap (bs) replicates. The Maximum Likelihood analysis was implemented in the graphical user interface raxmlGUI v7.4.2 [27]. Phylogram trees were visualized with Figtree v1.4.2 (http://tree.bio.ed.ac.uk/software/figtree/).

**Table 1:** GenBank sequences included in molecular analyses that belong to molecular operation taxonomic unit (MOUT) of *Saprolegnia* sp. 2 (*Saprolegnia aenigmatica* sp. nov) [12].

Designated name	Accession number	Strain	Host	Country	Publications generation the sequences
*Leptolegnia* sp.	AY310502	CBS 177.86			Oidtmann et al. 2004
*S*. *diclina*	DQ393513	UNCW217		USA	Hulvey et al. 2007
*Saprolegnia* sp.	DQ393514	UNCW218		USA	Hulvey et al. 2007
*Saprolegnia* sp.	DQ393515	UNCW219		USA	Hulvey et al. 2007
*Saprolegnia* sp.	DQ393517	UNCW250		USA	Hulvey et al. 2007
*Saprolegnia* sp.	DQ393539	UNCW290		USA	Hulvey et al. 2007
*Leptolegnia* sp.	EU240098	K08		Poland	Cordier et al. Unpublished
*Saprolegnia* sp.	EU348371	EM14	*Lithobates sphenocephalus*, egg	USA	Ruthig 2008
*Saprolegnia* sp.	EU348372	EM26	*Lithobates sphenocephalus*, egg	USA	Ruthig 2008
*Saprolegnia* sp.	EU480454	RACL2005	*Rana clamitans*, egg	USA	Karraker & Ruthig 2009
*Leptolegnia* sp.	FN186033	VI04813		United Kingdom	Stueland et al. Unpublished
*Achlya* sp.	GU014261	SCAAD		USA	Ruthig & Provost-Javier 2012
*Achlya* sp.	GU014262	CORA1		USA	Ruthig & Provost-Javier 2012
*Achlya* sp.	GU014263	EM31B	*Lithobates catesbeianus*, egg	USA	Ruthig & Provost-Javier 2012
*Achlya* sp.	GU014264	EM31E	*Lithobates catesbeianus*, egg	USA	Ruthig & Provost-Javier 2012
*Achlya* sp.	GU014265	EM26B	*Lithobates catesbeianus*, egg	USA	Ruthig & Provost-Javier 2012
*Achlya* sp.	GU014266	EM7	*Lithobates catesbeianus*, egg	USA	Ruthig & Provost-Javier 2012
*Achlya* sp.	GU014267	EM25		USA	Ruthig & Provost-Javier 2012
*Achlya* sp.	GU014268	DG		USA	Ruthig & Provost-Javier 2012
*Achlya* sp.	GU014269	PCRTB2	*Pseudacris crucifer*, egg	USA	Ruthig & Provost-Javier 2012
*Achlya* sp.	GU014270	MOTAD3		USA	Ruthig & Provost-Javier 2012
*Achlya* sp.	GU014271	O3EG1	*Lithobates catesbeianus*, egg	USA	Ruthig & Provost-Javier 2012
*Achlya* sp.	GU014272	EM23	*Lithobates catesbeianus*, egg	USA	Ruthig & Provost-Javier 2012
*Achlya* sp.	GU014273	EM32C	*Lithobates catesbeianus*, egg	USA	Ruthig & Provost-Javier 2012
*Achlya* sp.	GU014274	EM47A	*Lithobates catesbeianus*, egg	USA	Ruthig & Provost-Javier 2012
*Achlya* sp.	GU014275	MOT2		USA	Ruthig & Provost-Javier 2012
*Achlya* sp.	GU014276	02RCT	*Lithobates catesbeianus*, egg	USA	Ruthig & Provost-Javier 2012
*S*. *parasitica*	HQ644000	CBS 540.67		United Kingdom	Robideau et al. 2011, M.W. Dick
*Saprolegnia* sp.	JQ974985	LR11	*Rana cascadae*, egg	USA	Ault et al. 2012
*Saprolegnia* sp.	JQ974986	LB52	*Anaxyrus boreas*, egg	USA	Ault et al. 2012
*Saprolegnia* sp.	JQ974987	MB35/MB13	*Anaxyrus boreas*, egg	USA	Ault et al. 2012

Intra-specific and interspecific distances and number of ribotypes were calculated using only the sequences obtain for RJB-CSIC culture collection. These analyses were carried out using the program Geneious v6.14 [23].

### Nomenclature

The electronic version of this article in Portable Document Format (PDF) in an ISSN or ISBN publication is in accordance with the International Code of Nomenclature for algae, fungi, and plants. Hence new species names in the electronic publication of a PLoS ONE article are considered published under the ICN rules and printed copies are no longer required. Our new species names have been submitted to MycoBank and assigned a number, so by appending the number to the prefix http://www.mycobank.org/MB/, details can be accessed through any standard web browser. The online version of this work is archived and available from the following digital repositories: PubMed Central, and LOCKSS.

## Results

### Molecular and phylogenetic analysis

**Fig 1:**
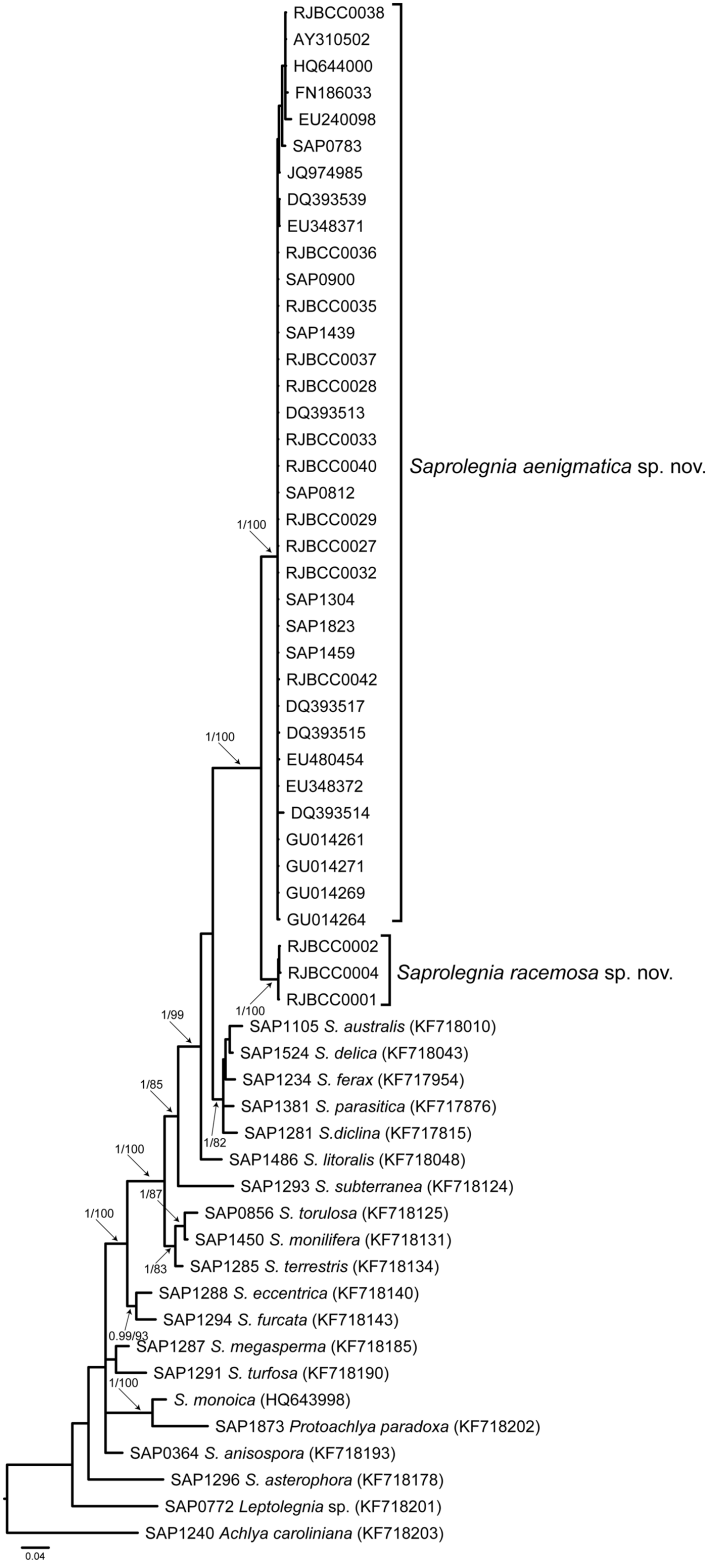
Phylogenetic relationships of *Saprolegnia aenigmatica* sp. nov and *Saprolegnia recemosa* sp. nov based on ITS rDNA. Phylogenetic tree was obtained from Bayesian inference analysis based on ITS rDNA sequences. Phylogenetic tree show the relationships among *S*. *aenigmatica*, *S*. *racemosa* and related *Saprolegnia* species. The numbers the branches represent the probability values (>0.95) and bootstrap support (> 75) obtained from Bayesian inference and Maximum Likelihood analyses respectively. The analyses comprise reference sequences of genus *Saprolegnia* [12] and all *Saprolegnia* isolates obtained in this study.

With all ITS nrDNA sequences an alignment matrix of 761 characters was generated. The phylogenetic analyses confirmed that sequences of isolates belonging to two new species grouped into two sister clades with high bootstrap values and posterior probabilities (pp = 1.0, bs = 100) (Fig 1). These clades were related to *Saprolegnia australis*, *S*. *delica*, *S*. *diclina*, *S*. *ferax*, *S*. *parasitica* and *S*. *litoralis* (Fig 1). The first clade included 116 sequences (85 from RJB culture collection, and 31 from GenBank). All sequences belonged to MOTU *Saprolegnia* sp. 2 (*i*.*e*., *S*. *aenigmatica*). The second clade included 19 sequences (none from GenBank) and all belonged to MOTU *Saprolegnia* sp. 3 (*i*.*e*., *S*. *racemosa*). A total of 21 ribotypes were identified in these two clades (18 for *S*. *aenigmatica*, and three for *S*. *racemosa*) (Table 2).

**Table 2:** Number of ribotypes, number of sequences per ribotype, and variable positions of ITS rDNA of *Saprolegnia aenigmatica* sp. nov. and *Saprolegnia racemosa* sp. nov. Variable positions of ITS rDNA sequences for other ribotypes are indicated in lowercase.

Ribotype	Number of sequences	Position in ITS1 region															Position in ITS2 region																												
		32	37	40	44	50	63	85	118	119	120	122	134	140	141	150	327	336	350	357	363	390	392	423	426	445	472	478	482	517	527	539	557	580	583	585	587	590	592	593	596	615	627	645	646
RJBCC0038^1^	21	**C**	**A**	**T**	**G**	**C**	**T**	**C**	**C**	**C**	**A**	**C**	**A**	**T**	**G**	**C**	**T**	**G**	**T**	**G**	**T**	**T**	**T**	**G**	**C**	**T**	**T**	**A**	**C**	**C**	**T**	**T**	**C**	**C**	**G**	**A**	**G**	**T**	**T**	**C**	**G**	**C**	**C**	**T**	**C**
RJBCC0027^1^	14																			a	c							g	t	t			t									t			
SAP0812^1^	11						c													a	c							g	t	t			t									t			
RJBCC0028^1^	8						c													a	c								t	t			t									t			
SAP0783^1^	5					t	c																			a		g		t															
SAP0900^1^	4																			a	c								t	t			t									t			
RJBCC0029^1^	4						y													a	c							g	y	t			t									t			
RJBCC0037^1^	3						c													a	c							r	t	t			t									t			
RJBCC0032^1^	3																			a	c							r	y	t			t									t			
RJBCC0035^1^	2						y													a	c							r	t	t			t									t			
RJBCC0042^1^	2						c													a	c							g		t			y									t			
RJBCC0040^1^	2						c													a	c							g		t			t									t			
SAP1304^1^	1																			a	c							g	y	t			t									t			
RJBCC0033^1^	1						y													a	c							g	y	t			t									t			
RJBCC0036^1^	1																			a	c							r	t	t			t									t			
SAP1439^1^	1						y													a	c								t	t			t									t			
SAP1459^1^	1																y			a	c								y	t			t									t			
SAP1823^1^	1																			a	c							g		t			t									t			
RJBCC0001^2^	13	**T**	**G**	**G**	**T**	**C**	**T**	**A**	**A**	**T**	**T**	**T**	**G**	**A**	**A**	**T**	**T**	**A**	**G**	**A**	**T**	**C**	**C**	**A**	**T**	**T**	**C**	**A**	**C**	**T**	**C**	**C**	**T**	**G**	**A**	**G**	**A**	**G**	**C**	**T**	**A**	**T**	**T**	**C**	**A**
RJBCC0002^2^	4																																	k											
RJBCC0004^2^	2																																	t											

^1^ *Saprolegnia aenigmatica*

^2^ *Saprolegnia racemosa*

The interspecific genetic distance between these two clades based on 653 characters was of 94.4% (± 0.2), and the number of variable positions between ribotypes of these two clades was 44 (Table 2). A total of 15 variable positions were present in the ITS1 region, and 29 in the ITS2 region (Table 2). The intraspecific genetic distance within the *S*. *aenigmatica* clade was 99.6% (± 0.4) (two variable positions in the ITS1 region and nine variable positions in the ITS2 region) (Table 2), while the intraspecific genetic distance within the *S*. *racemosa* clade was 99.9% (± 0.1) (one nucleotide change in the ITS2 region) (Table 2).

### Morphological character preservation

**Fig 2:**
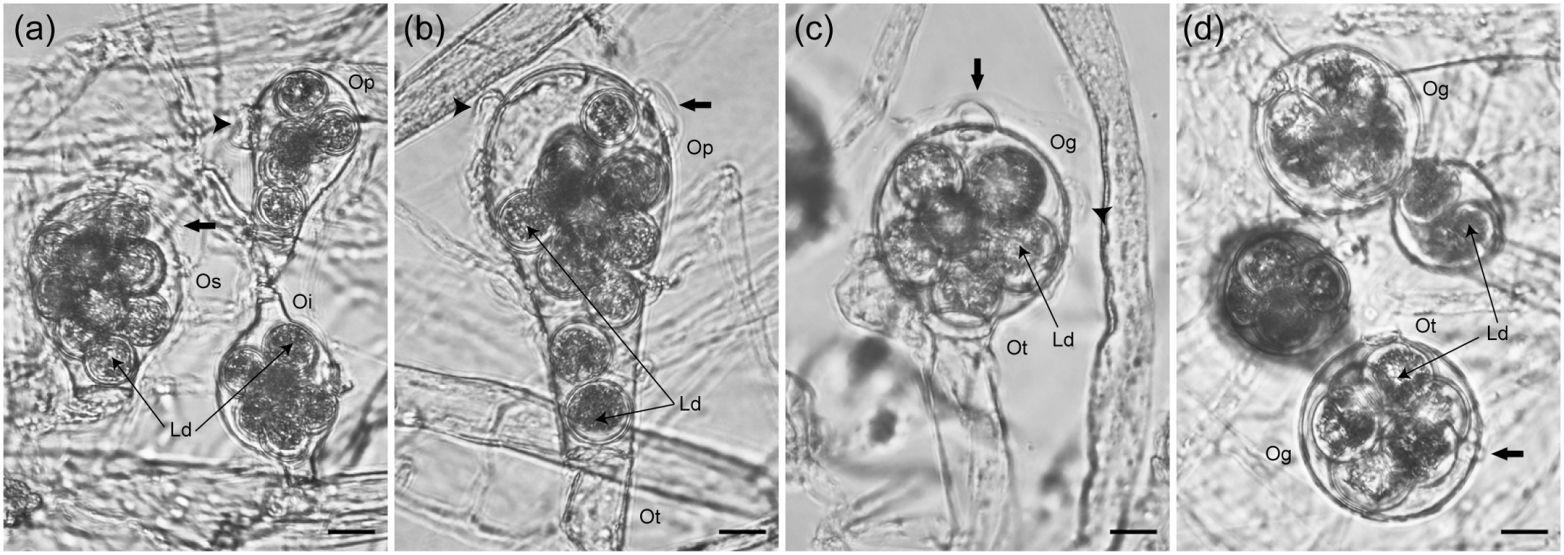
Preservation of morphological characters of *Saprolegnia* in absolute ethanol. The figure shows that the specific morphological features of *S*. *aenigmatica* (a and b) and *S*. *racemosa* (c and d) maintained after 12 months in absolute ethanol: oogonia globose (**Og**), oogonia subglobose (**Os**), oogonia pyriform (**Op**), oogonial stalk terminal (**Ot**), oogonial stalk intercalary (**Oi**), antheridial hypha (**arrow**). Compared to fresh material the antheridia attached to the oogonium walls were sometimes collapsed (**arrowhead**), and lipid droplets in the oospore became disrupted (**Ld**) preserved saples in ethanol. Bar = 20 μm.

After preservation in absolute ethanol for 12 months, oogonia stalks, number and shape of oospores, and antheridial branch origin, did not vary from those measured in fresh samples (Fig 2 and S1 Fig). The only differences observed were in the antheridia and lipid droplet positions in the oospores (Fig 2). In preserved samples, some of the antheridia attached to the oogonium walls were occasionally found to be collapsed, and oospore lipid droplets were always disrupted (Fig 2).

### Characterization of morphological features

Isolates from both clades produced sporangia, zoospores and cysts at all temperatures tested (Table 3). In the *S*. *aenigmatica* clade, only 7 out 23 isolates (35%) formed sexual structures (Table 3). The following isolates produced these structures at temperatures of 5, 10 and 15°C: RJBCC0024, RJBCC0026, RJBCC0027, RJBCC0028, RJBCC0032, RJBCC0038, and RJBCC0039. These isolates produced sporadic gemmae at all tested temperatures. The isolates RJBCC0020, RJBCC0021, RJBCC0022, RJBCC0023, RJBCC0025, RJBCC0029, RJBCC0030, RJBCC0031, RJBCC0033, RJBCC0034, RJBCC0035, RJBCC0036, RJBCC0037, RJBCC0040, RJBCC0041, and RJBCC0042 did not produce sexual structures, *i*.*e*., oogonia and antheridia, and instead produced abundant gemmae at all tested temperatures (Table 3). In the *S*. *racemosa* clade, all isolates produced sexual structures. Oogonia and antheridia were abundant at incubation temperatures between 5, 10, and 15°C, while at 20 and 25°C these were produced sporadically. Abundant gemmae were produced at 20 and 25°C, but were only occasionally produced at temperatures of 5, 10 and 15°C (Table 3).

**Table 3:** Summary of production of gemmae, oogonia, sporangia, zoospores and cyst of *Saprolegnia aenigmatica* sp. nov and *Saprolegnia racemosa* sp. nov. tested at selected temperatures. (+++) indicates that production was always observed, (++) indicates that production was often observed, and (+) indicates that production was rarely observed. Empty boxes indicate that no production at that temperature.

Species	Isolate	Gemmae					Oogonia				
		5°C	10°C	15°C	20°C	25°C	5°C	10°C	15°C	20°C	25°C
*Saprolegnia aenigmatica*	RJBCC0020	++	+++	+++	+++	+++					
	RJBCC0021	++	+++	+++	+++	+++					
	RJBCC0022	++	+++	+++	+++	+++					
	RJBCC0023	++	+++	+++	+++	+++					
	RJBCC0024	+	+	+	++	++		+++	+++		
	RJBCC0025	+	+++	+++	+++	++					
	RJBCC0026	+	+	+	+	+	+++	+++	+++	++	+
	RJBCC0027	+	+	+	+	+		++	+++	++	+
	RJBCC0028	+	+	+	+	+		+++	+++	+	
	RJBCC0029	++	++	++	+++	++					
	RJBCC0030	+	+	+++	+++	+++					
	RJBCC0031	++	+++	+++	+++	+++					
	RJBCC0032	+	+	+	+	+	+	+	+++	+	
	RJBCC0033	++	+++	+++	+++	+++					
	RJBCC0034	++	+++	+++	+++	+++					
	RJBCC0035	+	+	+++	+++	+++					
	RJBCC0036	++	+++	+++	+++	+++					
	RJBCC0037	++	+++	+++	+++	+++					
	RJBCC0038	+	+	+	+	+	++	+++	+++	+	
	RJBCC0039	+	+	+	+	+	+	+++	+++		
	RJBCC0040	++	+++	+++	+++	+++					
	RJBCC0041	++	+++	+++	+++	+++					
	RJBCC0042	++	+++	+++	+++	+++					
*Saprolegnia racemosa*	RJBCC0001	+	+	+	+++	+++	+++	+++	+++	+	+
	RJBCC0002	+	+	+	+++	+++	+++	+++	+++	+	+
	RJBCC0003	+	+	+	+++	+++	+++	+++	+++	+	+
	RJBCC0004	+	+	+	+++	+++	+++	+++	+++	+	+
	RJBCC0006	+	+	+	+++	+++	+++	+++	+++	+	+
	RJBCC0013	+	+	+	+++	+++	+++	+++	+++	+	+
	RJBCC0017	+	+	+	+++	+++	+++	+++	+++	+	+

**Fig 3:**
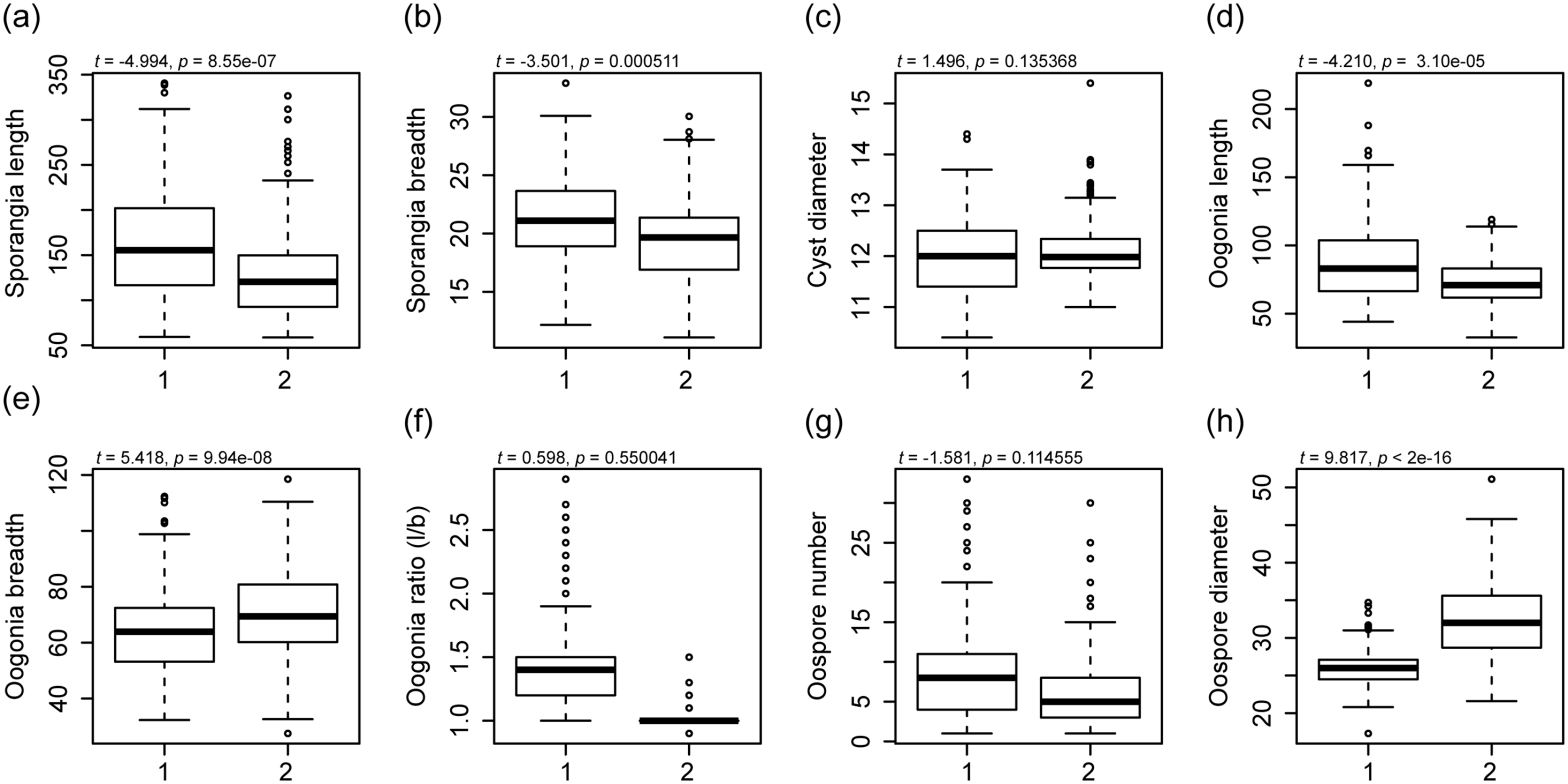
Boxplots representation of morphological features of *Saprolegnia aenigmatica* sp. nov. and *Saprolegnia racemosa* sp. nov. A linear model analysis shows that the whole set of features analyzed could differentiate *S*. *aenigmatica* (1) from *S*. *racemosa* (2) (*F*_8,441_ = 98.31, *p* < 0.001). The following morphological features contributed were considered: (a) sporangia length, (b) sporangia breadth, (d) oogonia length, (e) oogonia breadth, and (h) oospore diameter, while (c) cyst diameter, (f) oogonia ratio (l/b), and (g) oospore number did not contribute to differentiate *Saprolegnia* species.

**Fig 4:**
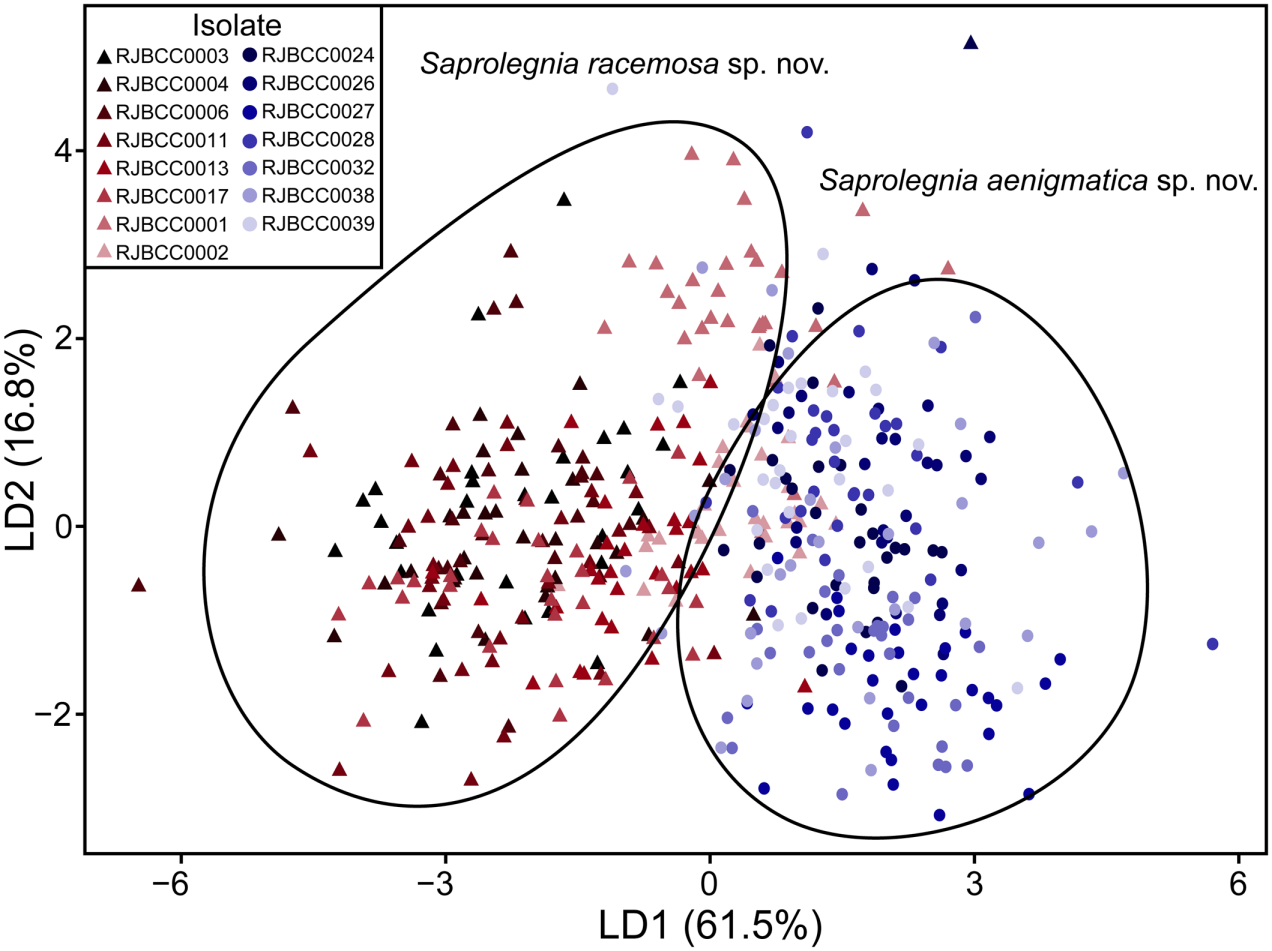
Discriminant linear function plot of morphological features of *Saprolegnia aenigmatica* sp. nov. and *Saprolegnia racemosa* sp. nov. The morphological features (sporangia length, sporangia breadth, cyst diameter, oogonia length, oogonia breadth, oogonia ratio (l/b), oospore number and oospore diameter) per isolate were used to separate *S*. *aenigmatica* (circles) from *S*. *racemosa* (triangles). Linear dimension 1 (LD1) explained 61.5% and linear dimension 2 (LD2) explained 16.8% of variance. The percentage of correct identifications based on measurements of morphological characters was 92.38% for isolates of the *S*. *aenigmatica* and 94.17% for *S*. *racemosa*.

The data on morphological characters are summarized in Table 4. Linear model analysis of the whole set of sexual and asexual characters showed significant differences between the two species (*F*_8,441_ = 98.31, *p* < 0.001). Individually, the following morphological characters were significantly different between species: sporangia length (*t* = -4.994, *p* < 0.001), sporangia breadth (*t* = -3.501, *p* < 0.001), oogonia length (*t* = -4.210, *p* < 0.001), oogonia breadth (*t* = 5.418, *p* < 0.001), and oospore diameter (*t* = 9.817, *p* < 0.001) (Fig 3). Data on cyst diameter, oogonia ratio, and the number of oospores per oogonium were not significantly different between the two clades (Fig 3). Thus, a linear discriminant analysis on morphological data could discriminate isolates from two clades. Linear dimension 1 (LD1) and linear dimension 2 (LD2) explained 61.5% to 78.3% of variance (Fig 4). The percentage of correct identifications based on measurements of morphological characters was 92.38% for isolates of *S*. *aenigmatica*, while in *S*. *racemosa*, the correct identifications were 94.17% (Fig 4).

**Table 4:** Measurements of sporangia, cysts, oogonia, and oospores, and antheridia type of *Saprolegnia aenigmatica* sp. nov. and *Saprolegnia racemosa* sp. nov.

	*Saprolegnia aenigmatica*							*Saprolegnia racemosa*							
	RJB0024	RJB0026	RJB0027	RJB0028	RJB0032	RJB0038	RJB0039	RJB0001	RJB0002	RJB0003	RJB0004	RJB0006	RJB0011	RJB0013	RJB0017
**Sporangia**															
Length mean (l)	179.8±34.3	228.9±62.7	198.4±33.0	146.7±60.2	147.0±46.1	124.4±35.4	124.9±42.4	90.0±16.7	110.3±23.0	125.9±39.6	131.7±39.2	122.0±45.6	144.9±56.2	142.8±41.5	171.6±60.2
Length range	122.8−270.2	136.7–340.6	110.6–239.4	59.3–283.7	74.1–253.1	69.8–229.9	61.6–239.2	58.7–141.9	64.9–164.7	72.4–222.2	74.0–232.7	70.0–326.5	66.3–311.7	77.6–265.8	81.0–300.2
Breadth mean (b)	19.3±2.2	24.5±3.0	21.4±2.4	23.5±3.4	21.0±2.7	20.2±3.7	20.0±2.3	20.0±2.0	18.0±2.7	18.3±3.5	20.7±3.8	18.9±2.7	18.4±4.6	20.1±3.0	20.1±3.3
Breadth range	14.5−24.2	18.4–30.1	16.5–27.5	18.0–32.9	15.7–27.2	12.2–28.3	16.2–25.3	14.0–23.3	14.2–23.3	11.9–23.9	11.2–30.1	14.0–25.4	11.1–28.1	11.5–28.7	15.1–27.3
**Cyst**															
Mean	12.3±0.8	12.7±0.6	11.3±0.3	11.8±0.5	11.3±0.4	11.9±0.6	12.3±0.6	13.0±0.5	12.0±0.3	12.2±0.5	12.0±0.3	12.2±0.6	11.9±0.3	11.9±0.3	11.9±0.2
diam range	11.4−15.4	11.6–14.3	10.4–11.9	11.0–13.1	10.6–13.0	10.9–13.5	11.2–14.4	11.5–13–9	11.6–13.1	11.4–13.9	11.6–12.7	11.0–13.8	11.1–12.7	11.3–12.8	11.6–12.3
**Oogonia**															
Length mean (l)	87.2±23.4	97.3±36.5	96.9±30.8	83.7±35.5	80.6±20.8	89.8±26.4	94.9±38.6	70.2±11.4	69.4±19.4	71.9±14.6	76.3±18.8	76.0±16.3	73.6±15.1	71.5±14.0	74.7±14.8
Length range	57.0−155.7	39.9–205.7	54.1–188.0	44.0–219.0	45.2–138.5	46.9–165.9	45.0–159.0	43.6–87.9	32.5–107.3	45.8–101.1	48.9–119.0	50.6–104.0	44.0–97.1	49.6–111.1	53.3–106.8
Breadth mean (b)	65.7±14.9	59.5±16.8	69.3±18.0	59.1±11.8	59.7±12.3	55.5±10.1	68.8±20.2	68.7±12.1	64.6±17.6	69.5±14.1	74.6±18.1	75.7±16.5	72.8±15.1	69.8±12.5	72.3±13.0
Breadth range	36.0−98.8	36.6–114.3	36.8–110.1	32.3–80.7	33.2–85.8	36.3–76.3	39.5–112.3	37.7–88.9	27.5–107.6	44.0–97.0	48.5–118.5	50.3–108.2	42.5–97.8	49.0–100.5	52.5–98.4
l/b ratio mean	1.4±0.3	1.6±0.3	1.4±0.2	1.4±0.4	1.4±0.4	1.6±0.4	1.4±0.3	1.0±0.1	1.1±0.1	1.0±0.0	1.0±0.0	1.0±0.0	1.0±0.0	1.0±0.0	1.0±0.1
l/b ratio range	1.0−2.3	1.1–2.2	1.0–1.7	1.0–2.7	1.0–2.5	1.2–2.9	1.0–2.3	0.9–1.2	1.0–1.3	1.0–1.1	1.0–1.1	1.0–1.1	1.0–1.2	0.9–1.1	0.9–1.3
**Oospores**															
Number mean	9.1±6.0	8.4±7.4	12.0±8.2	6.8±5.0	9.0±4.6	7.2±4.6	9.8±7.0	8.8±4.2	8.5±6.8	4.0±2.2	6.3±5.5	6.9±5.6	5.0±3.0	6.3±3.5	5.5±3.1
Number range	2.0−29.0	1.0–35.0	2.0–33.0	1.0–27.0	2.0–20.0	1.0–20.0	1.0–29.0	2.0–18.0	1.0–30.0	1.0–10.0	1.0–25.0	1.0–23.0	1.0–11.0	2.0–20.0	1.0–15.0
Mean diam	25.5±1.4	25.4±1.5	24.6±2.0	26.0±1.8	25.2±1.8	26.9±2.9	27.1±2.2	26.4±2.0	27.6±2.3	34.6±3.7	35.0±3.8	34.8±4.5	33.4±3.4	30.9±2.7	34.0±3.9
diam range	23.0−28.2	22.9–29.9	20.8–29.8	21.7–28.7	21.4–28.7	17.3–31.2	23.0–34.2	22.7–32.3	21.6–35.8	27.6–41.1	26.6–45.8	29.1–51.3	27.5–41.4	25.0–38.3	27.0–43.8
**Antheridia type**															
Diclinous	100%	100%	100%	100%	100%	100%	100%	98%	97%	98%	99%	99%	98%	98%	98%
Monoclinous								2%	3%	2%	1%	1%	2%	2%	2%

### Taxonomy

**Fig 5:**
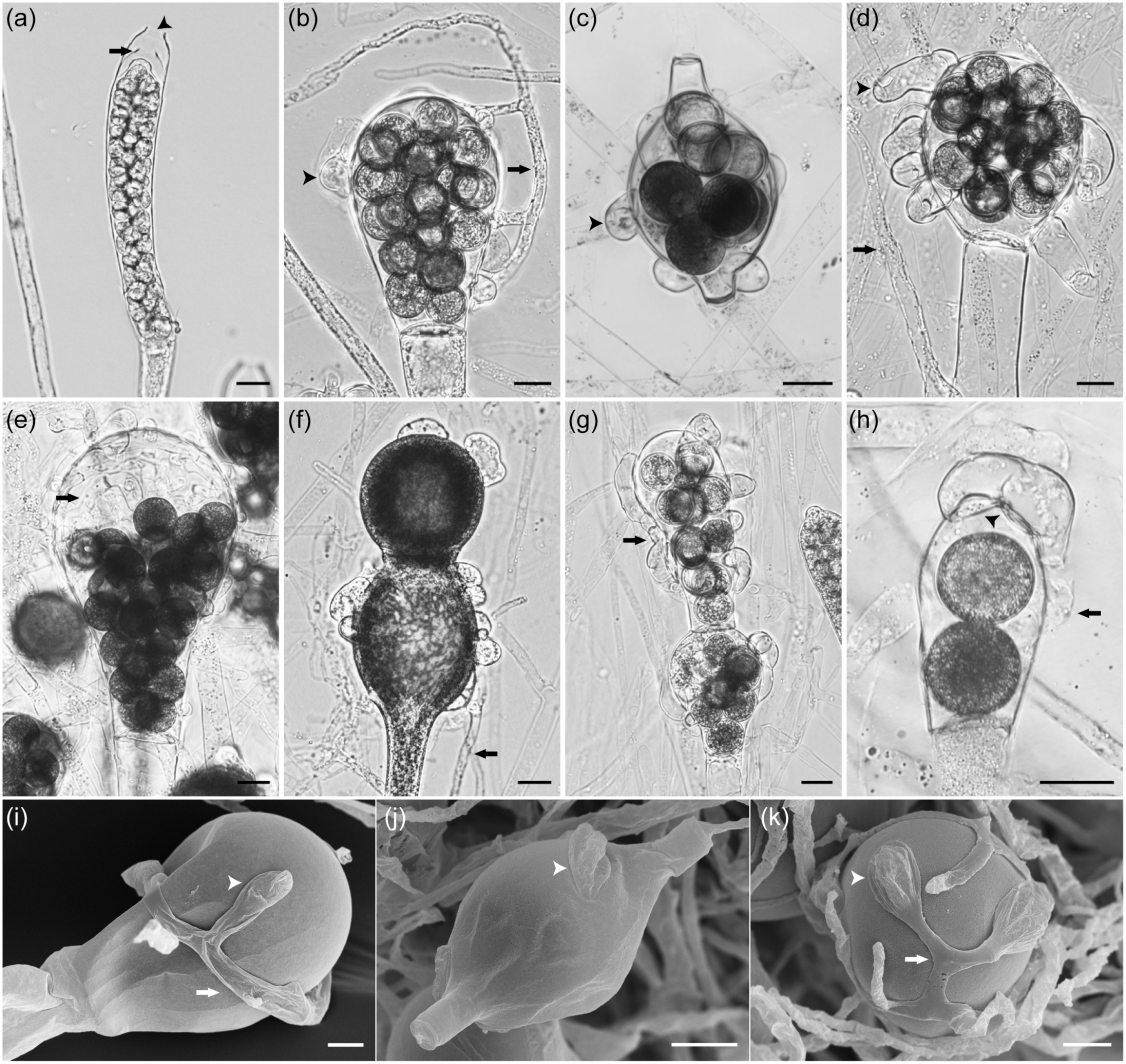
Specific morphological features of asexual and sexual structures of *Saprolegnia aenigmatica* sp. nov. Light (a, b, c, d, e, f, g, and h) and electronic micrographs (i, j, and k) of: (a) sporangia clavate, terminal, with internal proliferation (**arrows**) and apical papilla (**arrowhead**) (RJBCC0020); (b) oogonia pyriform, diclinous antheridial hyphae (**arrows**) well developed, branched, ampullaceous (**arrowhead**), and centric oospores (RJBCC0028); (c) oogonial intercalary, antheridial hyphae ampullaceous (**arrowhead**) and centric oospores (RJBCC0038); (d) oogonia subglobose, diclinous antheridial hyphae (**arrows**) well developed and from variable origin, tubular (**arrowhead**), and centric oospores (RJBCC0039); (e) oogonia pyriform, diclinous antheridial hyphae (**arrows**) from variable origin, encircling the oogonia, and centric oospores that do not fill the oogonium (RJBCC0024); (f) immature oogonia moniliform, and diclinous antheridial hyphae (**arrows**) from variable origin (RJBCC0027); (g) oogonia moniliform, and diclinous antheridial hyphae (**arrows**) from variable origin, encircling the oogonia (RJBCC0026); (h) oogonia pyriform, diclinous antheridial hyphae (**arrows**) well developed, attached by foot-like projections (**arrowhead**), and centric oospores (RJBCC0039); (i) oogonia pyriform, antheridial hyphae (**arrows**) well developed, encircling the oogonia, and antheridial attached (**arrowhead**) (RJBCC0024); (j) oogonial intercalary, antheridial hyphae attached (**arrowhead**) (RJBCC0028); (k) antheridial hyphae (**arrows**) well developed, encircling the oogonia, and antheridial attached (**arrowhead**) (RJBCC0028). Bar = 20 μm.

*Saprolegnia aenigmatica* Sandoval-Sierra & Diéguez-Uribeondo, *sp*. *nov*. (Figs 2a, 2b and 5).

MycoBank number: 812742.

#### Etymology

The specific epithet “*aenigmatica*” means that only a combination of morphological characters allows the identification of this species.

#### Diagnosis

This species can be distinguished from its closest relative, *S*. *racemosa*, based on the following morphological characters: sporangia length (164.3±59.0 9.) is larger than that of *S*. *racemosa* (129.9±48.3 μm), sporangia breadth (21.4±3.4 μm) is larger than that of *S*. *racemosa* (19.3±3.4 μm), oogonia length (88.6±29.6 μm) is larger than that of *S*. *racemosa* (73.0±15.9 μm), oogonia breadth (63.4±15.7 μm) is smaller than that of *S*. *racemosa* (71.0±15.4 μm), and oospore diameter (9.0±6.2 μm) is larger than that of *S*. *racemosa* (6.4±4.7 μm). This species can also be distinguished based on the sequence of ITS rDNA region.

#### Description

Hyphae of the vegetative mycelium are slender, delicate, aseptate, smooth, moderately branched. Gemmae, when present, generally branched, simple, catenulate; spherical, pyriform, irregular; often terminal. Sporangia clavate, rarely filiform; 164.3±59.0 × 21.4±3.4 μm (overall range 59.3–340.6 × 12.2–32.9 μm); mostly terminal, renewal usually by internal proliferation, with an apical papilla before zoospore discharge. Zoospore discharge saprolegnoid. Primary zoospores pyriform and apical biflagellate. Primary cysts sphaerical, 12.0±0.8 μm. (overall range 10.4–15.4 μm) in diameter. Secondary zoospores reniform and lateral biflagellate. Secondary cysts morphologically identical to primary cysts. Oogonia abundant when are present, globose, subglobose, pyriform and sometimes forming short moniliform chains, 88.6±29.6 × 63.4±15.7 μm (overall range 44.0–219.0 × 32.3–112.3 μm), the length/breadth ratio averaged 1.4±0.3 μm (overall range 1.0–2.9 μm). Oogonial wall unpitted, except under points of attachment of antheridial cells. Oogonia axillary, terminal, and intercalary. Oospores mostly filling the oogonium, but in some case do not filling the oogonium, globose to subglobose, 1–33 (9.0±6.2) in number; 25.6±2.3 μm (overall range 17.3–34.2 μm) in diameter. The internal structures of alive oospores are centric. Antheridial hyphae well developed, persistent, highly branched, of variable origin, always diclinous. Antheridia numerous, often encircling the oogonia, tubular to ampullaceous, sometimes branched, attached by foot-like projections.

#### Holotype

Specimen permanently preserved in a metabolically inactive state: parts preserved in absolute ethanol from single-spore isolate culture (marked as RJBCC0028) on 18^th^ Feb 2014, collected in Pucón, Chile, from *Salmo salar* egg in 2008 by J. Diéguez-Uribeondo & L. Zaror, MA-Fungi 88448; *ex-type*: RJBCC0028 of the Real Jardín Botánico culture collection; additional lyophilized material for DNA extraction is enclosed and marked together with the holotype. Isotype in UPS. GenBank accession number KR872872.

### Additional specimens examined

**Fertile isolates. Single-spore isolate cultures preserved in ethanol and lyophilized material from**: Spain, Ávila, Malpartida, from *Pelobates cultripes*, egg, 2007, M. Fernández-Benéitez, MA-Fungi 88456; culture strain RJBCC0024 of the Real Jardín Botánico culture collection; GenBank accession number KR872868. Spain, Ávila, Malpartida, from *Pelobates cultripes*, egg, 2007, M. Fernández-Benéitez, MA-Fungi 88457; culture strain RJBCC0026 of the Real Jardín Botánico culture collection; GenBank accession number KR872870. Chile, Pucón, from *Salmo salar*, alevin, 2008, J. Diéguez-Uribeondo & L. Zaror, MA-Fungi 88458; culture strain RJBCC0027 of the Real Jardín Botánico culture collection; GenBank accession number KR872871. Ecuador, Mindo, 2011, J. Diéguez-Uribeondo, MA-Fungi 88459; culture strain RJBCC0032 of the Real Jardín Botánico culture collection; GenBank accession number KR872876. France, La Llagonne, 7^th^ Jun 2012, J.V. Sandoval-Sierra, MA-Fungi 88460; culture strain RJBCC0038 of the Real Jardín Botánico culture collection; GenBank accession number KR872882. Spain, Cataluña, Avellanet, 10^th^ Jun 2012, J.V. Sandoval-Sierra, MA-Fungi 88461; culture strain RJBCC0039 of the Real Jardín Botánico culture collection; GenBank accession number KR872883.

**Sterile isolates. Single-spore isolate cultures from:** Argentina, 2010, M.M. Steciow; culture strain RJBCC0030 of the Real Jardín Botánico culture collection; GenBank accession number KR872874. Chile, Los Lagos (X Region), Palena province, Comuna Hualaihué, Comau fjord, Huinay Reserve, 17^th^ Nov 2012, J.V. Sandoval-Sierra; culture strain RJBCC0020 of the Real Jardín Botánico culture collection; GenBank accession number KR872864. Chile, Los Lagos (X Region), Palena province, Comuna Hualaihué, Comau fjord, Huinay Reserve, 17^th^ Nov 2012, J.V. Sandoval-Sierra; culture strain RJBCC0040 of the Real Jardín Botánico culture collection; GenBank accession number KR872884. Chile, Los Lagos (X Region), Palena province, Comuna Hualaihué, Comau fjord, Huinay Reserve, 17^th^ Nov 2012, J.V. Sandoval-Sierra; culture strain RJBCC0041 of the Real Jardín Botánico culture collection; GenBank accession number KR872885. Chile, Los Lagos (X Region), Palena province, Comuna Hualaihué, Comau fjord, Huinay Reserve, 19 Nov 2012, J.V. Sandoval-Sierra; culture strain RJBCC0042 of the Real Jardín Botánico culture collection; GenBank accession number KR872886. Ecuador, Morona Santiago, 2010, J.V. Sandoval-Sierra; culture strain RJBCC0029 of the Real Jardín Botánico culture collection; GenBank accession number KR872873. Ecuador, Morona Santiago, 2011, A. Alaminos; culture strain RJBCC0033 of the Real Jardín Botánico culture collection; GenBank accession number KR872877. Ecuador, Morona Santiago, 2011, A. Alaminos; culture strain RJBCC0034 of the Real Jardín Botánico culture collection; GenBank accession number KR872878. Ecuador, Morona Santiago, 2011, A. Alaminos; culture strain: RJBCC0035 of the Real Jardín Botánico culture collection; GenBank accession number KR872879. Ecuador, Morona Santiago, 2011, A. Alaminos; culture strain RJBCC0036 of the Real Jardín Botánico culture collection; GenBank accession number KR872880. Ecuador, Morona Santiago, 2011, A. Alaminos; culture strain RJBCC0037 of the Real Jardín Botánico culture collection; GenBank accession number KR872881. Spain, Avila, Malpartida, from *Pelobates cultripes* egg, 2007, M. Fernández-Benéitez; culture strain RJBCC0025 of the Real Jardín Botánico culture collection; GenBank accession number KR872869. Spain, Cataluña, Girona, from *Barbus meridionalis* caudal fin, 25^th^ Jan 2014; culture strain RJBCC0022 of the Real Jardín Botánico culture collection; GenBank accession number KR872866. Spain, Doñana Biological Station, 2007, J. Diéguez-Uribeondo; culture strain RJBCC0023 of the Real Jardín Botánico culture collection; GenBank accession number KR872867. Spain, Granada, Sierra Nevada, 3th May 2010, J.V. Sandoval-Sierra; culture strain RJBCC0031 of the Real Jardín Botánico culture collection; GenBank accession number KR872875. Spain, Navarra, Urtasun, 28^th^ Apr 2013, J.V. Sandoval-Sierra & S. Rezinciuc; culture strain: RJBCC0021 of the Real Jardín Botánico culture collection; GenBank accession number KR872865.

#### Distribution and host range

Currently known from Argentina, Chile, Czech Republic, Ecuador, France, Poland, Spain, United Kingdom, and the USA (S1 Table and Table 1). Substrates include eggs of fish (*Oncorhynchus mykiss* and *Salmo salar*), amphibians (*Anaxyrus boreas*, *Epidalea calamita*, *Hyloscirtus alytolylax*, *Lithobates catesbeianus*, *Pelobates cultripes*, *Pelophylax perezi*, *Pseudacris crucifer*, *Pseudacris regilla*, *Rana cascadae*, *Rana clamitans*, *Scinax garbei*), and an adult fish, *Barbus meridionalis* (S1 Table and Table 1).

#### Notes

In the GenBank some sequences of this species are deposited under the names *Achlya* sp., *S*. *diclina*, *S*. *parasitica*, and *Leptolegnia* sp. (Table 1).

**Fig 6:**
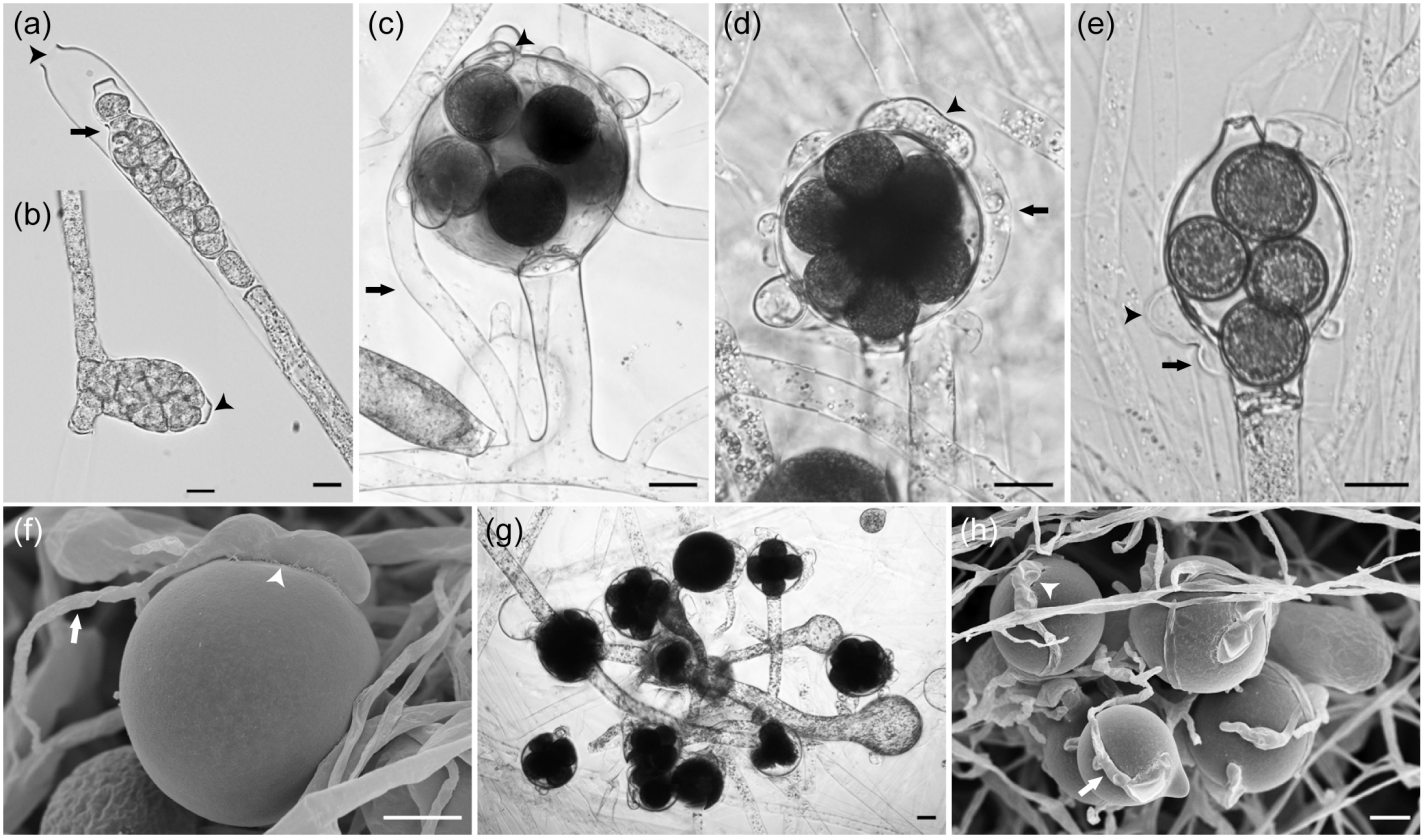
Specific morphological features of asexual and sexual structures of *Saprolegnia racemosa* sp. nov. Light (a, b, c, d, e and g) and electronic micrographs (f and h) of: (a) sporangia clavate, terminal, with internal proliferation (**arrows**) and apical papilla (**arrowhead**) (RJBCC0003); (b) sporangia apical and apical papilla (**arrowhead**) (RJBCC0011); (c) oogonia globose, monoclinous antheridial hyphae (**arrows**) well developed and from variable origin, ampullaceous (**arrowhead**), and centric oospores (RJBCC0003); (d) oogonia globose, diclinous antheridial hyphae (**arrows**) well developed, circling the oogonia, tubular (**arrowhead**), and centric oospores (RJBCC0001); (e) oogonial intercalary, antheridia ampullaceous (**arrows**), attached by foot-like projections (**arrowhead**), and centric oospores (RJBCC0017); (f) oogonia globose, diclinous antheridial hyphae (**arrows**) well developed, and attached by foot-like projections (**arrowhead**) (RJBCC0001); (g) oogonia globose, grouped like racemosa structure, and centric oospores (RJBCC0002); (h) oogonia globose, grouped like racemosa structure, diclinous antheridial hyphae (**arrows**) well developed, and attached by foot-like projections (**arrowhead**) (RJBCC0002). Bar = 20 μm.

*Saprolegnia racemosa* Sandoval-Sierra & Diéguez-Uribeondo, *sp*. *nov*. (Figs 2c, 2e and 6).

MycoBank number: 812743.

#### Etymology

The specific epithet “*racemosa*” refers to the oogonia arrangement, often adopting the form of a raceme.

#### Diagnosis

This species can be distinguished by the formation of oogonia racemes, which is a unique characteristic in the genus *Saprolegnia*, and also by the sequence of the ITS rDNA region.

#### Description

Hyphae of the vegetative mycelium are slender, delicate, aseptate, smooth, moderately branched. Gemmae, when are present, generally branched, catenulate when are intercalary and clavate when are terminal. Sporangia clavate, rarely filiform; 129.9±48.3 × 19.3±3.4 μm. (overall range 58.7–326.5 × 11.1–30.1 μm); mostly terminal, some times apical, renewal usually by internal proliferation, with an apical papilla before zoospore discharge. Zoospore discharge saprolegnoid. Primary zoospores pyriform and apical biflagellate. Primary cysts sphaerical, 12.1±0.5 μm (overall range 11.0–13–9 μm) in diameter. Secondary zoospores reniform and lateral biflagellate. Secondary cysts morphologically identical to primary cysts. Oogonia abundant, often grouped in racemose structures. Oogonia abundant, globose, rarely obovoid, 73.0±15.9 × 71.0±15.4 μm (overall range 32.5–119.0 × 27.5–118.5 μm), the length/breadth ratio averaged 1.0±0.1 μm (overall range 0.9–1.3 μm). Oogonial wall unpitted, except under points of attachment of antheridial cells. Oogonial stalks often branched to form a group, sometimes axillary and occasionally intercalary. Oospores mostly filling the oogonium, globose to subglobose, 1–30 (6.4±4.7) in number; 31.7±4.7 μm (overall range 21.3–51.3 μm) in diameter. The internal structures of alive oospores are centric. Antheridial hyphae well developed, persistent, of variable origin, mainly diclinous, sporadically monoclinous. Antheridia numerous, often circling the oogonia, tubular to ampullaceous, attached by foot-like projections.

#### Holotype

Specimen permanently preserved in a metabolically inactive state: parts preserved in absolute ethanol from single-spore isolate culture (marked as RJBCC0002), in 18^th^ Feb 2014, collected in Rascafría, Madrid, Spain, from *Pelophylax perezi* egg, 8^th^ May 2011, J.V. Sandoval-Sierra, MA-Fungi 88447; *ex-type*: RJBCC0002 of the Real Jardín Botánico culture collection; additional lyophilized material for DNA extraction is enclosed and marked together with the holotype. GenBank accession number KR872846.

#### Additional specimens examined

All locations are in Spain: Burgos, 5^th^ Nov 2010, J.V. Sandoval-Sierra, MA-Fungi 88449; culture strain RJBCC0001 of the Real Jardín Botánico culture collection; GenBank accession number KR872845. Ávila, Barco de Ávila, 7^th^ Aug 2014, J.V. Sandoval-Sierra, MA-Fungi 88450; culture strain RJBCC0003 of the Real Jardín Botánico culture collection; GenBank accession number KR872847. Ávila, Becedas, 7^th^ Aug 2014, J.V. Sandoval-Sierra, MA-Fungi 88451; culture strain RJBCC0004 of the Real Jardín Botánico culture collection; GenBank accession number KR872848. Ávila, Becedas, 7^th^ Aug 2014, J.V. Sandoval-Sierra, MA-Fungi 88452; culture strain RJBCC0006 of the Real Jardín Botánico culture collection; GenBank accession number KR872850. Ávila, Casa del Abad, 7^th^ Aug 2014, J.V. Sandoval-Sierra, MA-Fungi 88453; culture strain RJBCC0011 of the Real Jardín Botánico culture collection; GenBank accession number KR872855. Ávila, Gavilanes, 10^th^ Aug 2014, J.V. Sandoval-Sierra, MA-Fungi 88454; culture strain RJBCC0013 of the Real Jardín Botánico culture collection; GenBank accession number KR872857. Ávila, Villa Nueva de Ávila, 10^th^ Aug 2014, J.V. Sandoval-Sierra, MA-Fungi 88455; culture strain RJBCC0017 of the Real Jardín Botánico culture collection; GenBank accession number KR872861. All specimens examined were permanently preserved in a metabolically inactive state: parts preserved both lyophilized and in absolute ethanol from single-spore isolates.

#### Distribution and host range

Currently known only in Spain. Substrates include *Pelophylax perezi* egg.

## Discussion

Taxonomy is a comparative science and requires intensive and long-term study of many specimens and developmental stages of an organism rather than a casual and short-term examination of a few scraps [28, 29]. The publication of new species should be undertaken with great care [28]. In this study, we described two species, *Saprolegnia aenigmatica* sp. nov., and *Saprolegnia racemosa* sp. nov, previously designated as MOTUs *Saprolegnia* sp. 2 and *Saprolegnia* sp. 3, respectively [12]. We have applied a new multi-faceted approach that integrates the recently defined ITS rDNA-based MOTUs of *Saprolegnia* [12], their phylogenetic relationships, and the analyses of a set of morphological features. In addition, we have included in the protologues suitably preserved holotypes, such as specimens preserved in absolute ethanol, and moreover additional material such as lyophilized specimens, and *ex-types* as single-spore isolated pure cultures that ensure the possibility of further morphological comparisons and molecular analyses.

The phylogenetic analyses of these isolates confirmed the two new species are closely related. Comparisons of morphological characters of isolates from MOTU *Saprolegnia* sp 3, show a distinct character, *i*.*e*., “aggrupation of oogonia in racemes”, that seems to be species-specific, and has never been observed in other *Saprolegnia* species [1, 2, 30–37]. Therefore, this new species was named *S*. *racemosa*. Because specimens of *S*. *racemosa* easily produced sexual structures, and have this unique feature, they are easily recognizable. Isolates of *Saprolegnia* sp 2, however, have a high number (more than 60%) of sterile isolates, *i*.*e*., not forming sexual structures, and the features observed are not species-specific. This high percentage of sterile isolates has only been reported for *S*. *parasitica* [10, 38, 39]. Because of this problem, the isolates of clade *Saprolegnia* sp 2 were named *S*. *aenigmatica*.

In order to check the possibility of using morphological characters as a source of taxonomic value, we have considered not only the analyses of a single morphological feature, *i*.*e*., sporangia length and breadth, oogonia length and breadth, and oospore diameter, but also of a combination of them in a wide range of isolates. The analyses on isolates of both *S*. *aenigmatica* and *S*. *racemosa* indicated that both species could be distinguished using this set of morphological features. These analyses ought to be applied to the rest of *Saprolegnia* species in order to check their validity for species identification in the whole genus. This is important since several species of *Saprolegnia* have been described based only on data from a single isolate, and do not consider the possibility of data overlapping of morphological features [30–33, 35–37].

The lack of available and suitable holotypes does not only affect the possibility of studying morphological characters, but also, the possibility of performing DNA analyses. So far, the most reliable approach for classifying and identifying *Saprolegnia* appears to be the application of DNA sequence and phylogenetic analyses of informative DNA regions [12]. This is also the case for other groups of organisms such as green algae, red algae, diatoms, brawn algae, rotifers, peronosporales, insects, etc. [40–44]. Holotypes should provide enough information to build a robust taxonomy. Poor conditions of the holotypes are often the reason for the difficultly of taking decisions on assigning a new species [45]. Thus, in some morphological challenging groups, such as microalgae, most protologues currently take into consideration protocols for preserving holotypes by specimen cryopreservation [46]. The International Code of Nomenclature for algae, fungi, and plants (ICN) has recently incorporated specifications regarding particularities on holotype preservations [13, 46]. However, researches still have to deal with previous protologues and collections that do not specify the conditions of holotype preservation.

*Saprolegnia* is not an exception, and there is not any standardized protocol for suitable holotype preservation. Current methods used for preserving type *Saprolegnia* species do not guarantee, neither preserving the specific morphological data, nor their use for DNA analyses, or both (*e*.*g*., *Saprolegnia australis*) [47]. So far, the majority of holotypes in *Saprolegnia* and related genera are not suitable for morphological or molecular comparisons, and basically consists of illustrations from specimens, which are not useful for species comparison neither for description of new species. This could be solved by adding suitably preserved holotypes, but also with the incorporation of *ex-types* as pure cultures that will certainly help to solve this problem. Although *ex-types* are not accepted as nomenclatural types in the International Code of Nomenclature for algae, fungi, and plants [13], several *Saprolegnia* protologues have included *ex-types* since 1977, *i*.*e*., *S*. *shikotsuensis*, *S*. *polymorpha*, *S*. *longicaulis*, *S*. *semihypogyna*, *S*. *milnae*, *S*. *variabilis*, *S*. *oliviae*, *S*. *multispora* and *S*. *bulbosa* [30–37, 48]. The *ex-types* as pure cultures have the advantage of allowing DNA extractions, and also the reproduction of morphological features *de novo*. They are also crucial for key studies such as genomic, metabolomics, proteomic, etc., and greatly facilitates other traditional studies. On the contrary, the *ex-types* have the disadvantage that maintaining living organisms is difficult, costly, and that there is a possibility to loose them, *e*.*g*., the *ex-types* of *S*. *polymorpha* (*ex-type* CBS 618.97) [30], *S*. *longicaulis* (*ex-type* LPSC N° 633) [31], *S*. *milnae* (*ex-type* LPSC N° 739) [32], and *S*. *oliviae* (*ex-type* LPSC N° 746) [33]. Because of that, when possible, we suggest to maintain cultures in at least two culture collections to ensure its durability over time as proposed by [15].

Taking into account the existing methodological problems in *Saprolegnia* taxonomy and the progress of molecular taxonomy, in this study, we propose a standardized protocol that can increase the stability of nomenclature and facilitate building a robust taxonomy in this genus. The protocol consists of including holotypes preserved with methods that do not alter the specific morphological feature, and that can be subjected to DNA extractions, *i*.*e*., in absolute ethanol and/or by lyophilization. Moreover, we include the specific mention of pure cultures from single-spore isolates as *ex-types* in the protologues. Single-spore isolates ensure that characters, including reference sequences [49] or genotypes [50], arise from a single individual. Thus, we propose to: (i) incorporate, when possible, several isolates of the new species, which allow the description of the variation of morphological and molecular characters; (ii) generate ITS rDNA sequences and to compare them to *Saprolegnia* sequences from reference data-bases; (iii) perform phylogenetic analysis to check their relationships with other species; (iv) preserve holotypes in absolute ethanol to maintain the specific morphological characters, and incorporate additional lyophilized material to allow molecular analyses; (v) include *ex-type* as pure cultures from single-spore isolates that will facilitate reproducibility of characters under different conditions, and preserve the *ex-type* at least in two different collections.

Until now, there has not been formal standards for descriptions, but there are some internationally accepted requirements for proposing new names, which are defined and regulated by the International Code of Nomenclature for algae, fungi, and plants [13]. The approach proposed here allows the suitable preservation of the holotypes (*i*.*e*., of all the characters) satisfying all guidelines of the ICN. The description, identification and classification of organisms are the central column in biodiversity and evolutionary studies and require the generation of a robust taxonomy. This will ensure future studies in this genus that comprises important pathogens that are being reported as responsible for high-profiled declines in animal wildlife and aquaculture.

## Supporting Information

S1 Fig Boxplots representation of the preservation of morphological characters of *Saprolegnia aenigmatica* sp. nov. and *Saprolegnia racemosa* sp. nov. in absolute ethanol. The morphological features (oogonia length, oogonia breadth, oogonia ratio (l/b), oospore number and oospore diameter) were compared between fresh and preserved specimens of *S*. *aenigmatica* (a, c, e, g, and i) and *S*. *racemosa* (b, d, f, h, and j). The comparisons were implemented using one-way ANOVA analysis.
(TIF)

S1 Table Isolates of *Saprolegnia* sp. nov. included in the molecular analyses. Sequences were obtained from cultures of the RJB-CSIC collections. The sequences included in molecular analyses were designated according to molecular operation taxonomic units (MOTUs): *Saprolegnia* sp. 2 (*Saprolegnia aenigmatica* sp. nov.) and *Saprolegnia* sp. 3 (*Saprolegnia racemosa* sp. nov.). New sequences generated in this study are marked in bold.
(PDF)
