# Supplementary figures and images for: A Comprehensive Protocol for Improving the Description of Saprolegniales (Oomycota): Two Practical Examples (Saprolegnia aenigmatica sp. nov. and Saprolegnia racemosa sp. nov.)
Source: PLoS One. 2015 Jul 17;10(7):e0132999. doi: 10.1371/journal.pone.0132999 (PMC4506062; doi:10.1371/journal.pone.0132999)

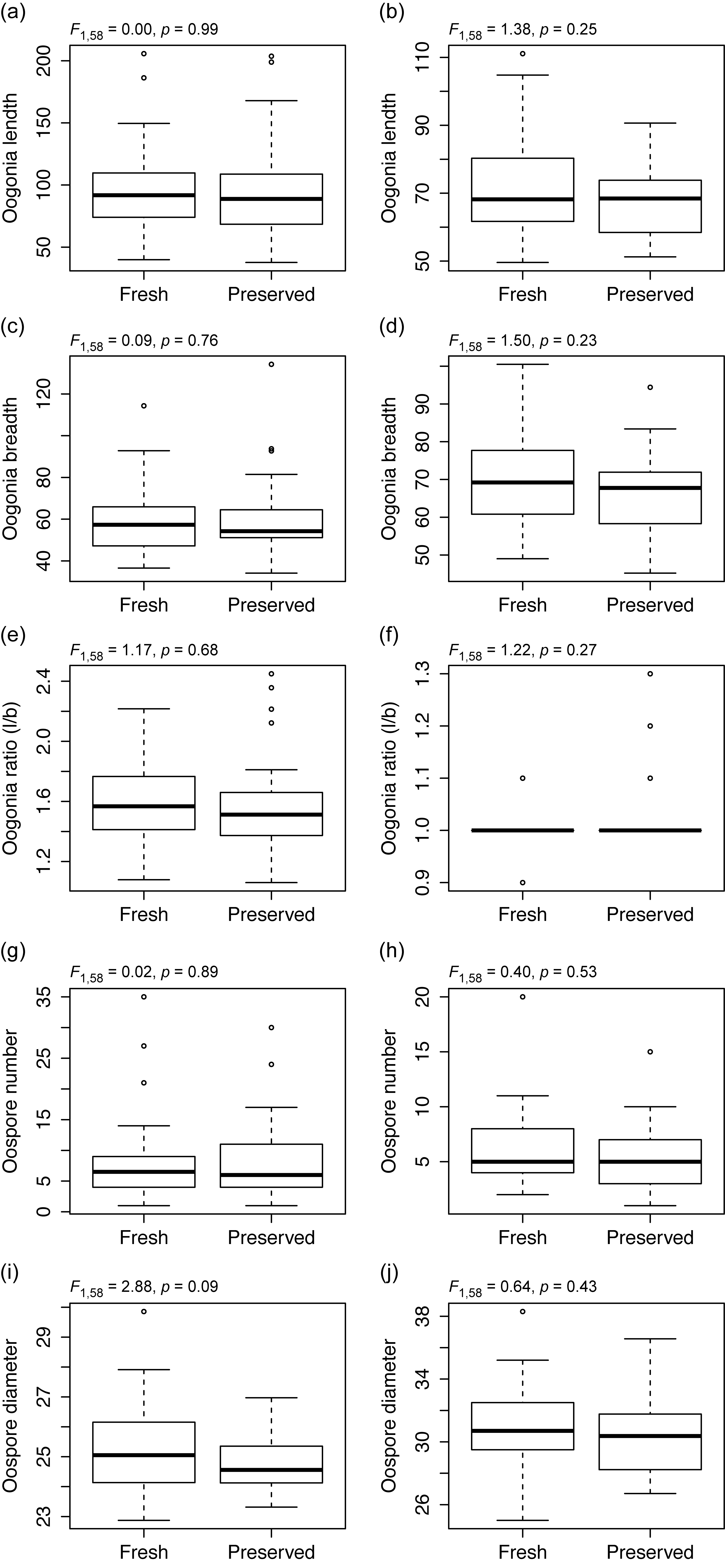

Supplement: S1 Fig — The morphological features (oogonia length, oogonia breadth, oogonia ratio (l/b), oospore number and oospore diameter) were compared between fresh and preserved specimens of S. aenigmatica (a, c, e, g, and i) and S. racemosa (b, d, f, h, and j). The comparisons were implemented using one-way ANOVA analysis. (TIF) [file pone.0132999.s001.tif]
