# Supplementary material for: A Comprehensive Protocol for Improving the Description of Saprolegniales (Oomycota): Two Practical Examples (Saprolegnia aenigmatica sp. nov. and Saprolegnia racemosa sp. nov.)
Source: PLoS One. 2015 Jul 17;10(7):e0132999. doi: 10.1371/journal.pone.0132999 (PMC4506062; doi:10.1371/journal.pone.0132999)
Supplement: S1 Table — Sequences were obtained from cultures of the RJB-CSIC collections. The sequences included in molecular analyses were designated according to molecular operation taxonomic units (MOTUs): Saprolegnia sp. 2 (Saprolegnia aenigmatica sp. nov.) and Saprolegnia sp. 3 (Saprolegnia racemosa sp. nov.). New sequences generated in this study are marked in bold. (PDF) [file pone.0132999.s002.pdf]

S1 Table. Isolates of *Saprolegnia* sp. nov. included in the molecular analyses. Sequences were obtained from cultures of the RJB-CSIC collections. The sequences included in molecular analyses were designated according to molecular operation taxonomic units (MOTUs): *Saprolegnia* sp. 2 (*Saprolegnia aenigmatica* sp. nov.) and *Saprolegnia* sp. 3 (*Saprolegnia racemosa* sp. nov.). New sequences generated in this study are marked in bold.

RJB[1]	MOTUs	Host/Habitat	Country and state/province	Year of isolation	Accession number
RJBCC0001	*Saprolegnia* sp. 3	River	Spain, Burgos	2010	**KR872845**
RJBCC0002	*Saprolegnia* sp. 3	*Pelophylax perezi*, egg	Spain, Madrid, Rascafria	2011	**KR872846**
RJBCC0003	*Saprolegnia* sp. 3	River	Spain, Avila, Barco de Avila	2014	**KR872847**
RJBCC0004	*Saprolegnia* sp. 3	River	Spain, Avila, Becedas	2014	**KR872848**
RJBCC0005	*Saprolegnia* sp. 3	River	Spain, Avila, Becedas	2014	**KR872849**
RJBCC0006	*Saprolegnia* sp. 3	River	Spain, Avila, Becedas	2014	**KR872850**
RJBCC0007	*Saprolegnia* sp. 3	River	Spain, Avila, Solana de Avila	2014	**KR872851**
RJBCC0008	*Saprolegnia* sp. 3	River	Spain, Avila, Solana de Avila	2014	**KR872852**
RJBCC0009	*Saprolegnia* sp. 3	River	Spain, Avila, Casa del Rey	2014	**KR872853**
RJBCC0010	*Saprolegnia* sp. 3	River	Spain, Avila, Casa del Rey	2014	**KR872854**
RJBCC0011	*Saprolegnia* sp. 3	River	Spain, Avila, Casa del Abad	2014	**KR872855**
RJBCC0012	*Saprolegnia* sp. 3	River	Spain, Avila, Gavilanes	2014	**KR872856**
RJBCC0013	*Saprolegnia* sp. 3	River	Spain, Avila, Gavilanes	2014	**KR872857**
RJBCC0014	*Saprolegnia* sp. 3	River	Spain, Avila, Mijares	2014	**KR872858**
RJBCC0015	*Saprolegnia* sp. 3	River	Spain, Avila, Mijares	2014	**KR872859**
RJBCC0016	*Saprolegnia* sp. 3	River	Spain, Avila, Mijares	2014	**KR872860**
RJBCC0017	*Saprolegnia* sp. 3	River	Spain, Avila, Villa Nueva de Avila	2014	**KR872861**
RJBCC0018	*Saprolegnia* sp. 3	River	Spain, Avila, Villa Nueva de Avila	2014	**KR872862**
RJBCC0019	*Saprolegnia* sp. 3	River	Spain, Avila, Navarrevisca	2014	**KR872863**
RJBCC0020	*Saprolegnia* sp. 2	River	Chile, Puerto Montt, Huinay Scientific Field Station	2012	**KR872864**
RJBCC0021	*Saprolegnia* sp. 2	River	Spain, Navarra, Urtasun	2013	**KR872865**
RJBCC0022	*Saprolegnia* sp. 2	*Barbus meridionalis*, caudal fin	Spain, Cataluña, Girona	2014	**KR872866**
RJBCC0023	*Saprolegnia* sp. 2	River	Spain, Doñana Biological Station	2007	**KR872867**
RJBCC0024	*Saprolegnia* sp. 2	*Pelobates cultripes*, egg	Spain, Avila, Malpartida	2007	**KR872868**
RJBCC0025	*Saprolegnia* sp. 2	*Pelobates cultripes*, egg	Spain, Avila, Malpartida	2007	**KR872869**

RJBCC0026	*Saprolegnia* sp. 2	*Pelobates cultripes*, egg	Spain, Avila, Malpartida	2007	**KR872870**
RJBCC0027	*Saprolegnia* sp. 2	*Salmo salar*, alevin	Chile, Pucón	2008	**KR872871**
RJBCC0028	*Saprolegnia* sp. 2	*Salmo salar*, egg	Chile, Pucón	2008	**KR872872**
RJBCC0029	*Saprolegnia* sp. 2	River	Ecuador, Morona Santiago	2010	**KR872873**
RJBCC0030	*Saprolegnia* sp. 2	River	Argentina	2010	**KR872874**
RJBCC0031	*Saprolegnia* sp. 2	River	Spain, Granada, Sierra Nevada	2010	**KR872875**
RJBCC0032	*Saprolegnia* sp. 2	River	Ecuador, Mindo	2011	**KR872876**
RJBCC0033	*Saprolegnia* sp. 2	River	Ecuador, Morona Santiago	2011	**KR872877**
RJBCC0034	*Saprolegnia* sp. 2	River	Ecuador, Morona Santiago	2011	**KR872878**
RJBCC0035	*Saprolegnia* sp. 2	Lake	Ecuador, Morona Santiago	2011	**KR872879**
RJBCC0036	*Saprolegnia* sp. 2	River	Ecuador, Morona Santiago	2011	**KR872880**
RJBCC0037	*Saprolegnia* sp. 2	River	Ecuador, Morona Santiago	2011	**KR872881**
RJBCC0038	*Saprolegnia* sp. 2	River	France, La Llagonne	2012	**KR872882**
RJBCC0039	*Saprolegnia* sp. 2	River	Spain, Cataluña, Avellanet	2012	**KR872883**
RJBCC0040	*Saprolegnia* sp. 2	River	Chile, Puerto Montt, Huinay Scientific Field Station	2012	**KR872884**
RJBCC0041	*Saprolegnia* sp. 2	River	Chile, Puerto Montt, Huinay Scientific Field Station	2012	**KR872885**
RJBCC0042	*Saprolegnia* sp. 2	River	Chile, Puerto Montt, Huinay Scientific Field Station	2012	**KR872886**
SAP0433	*Saprolegnia* sp. 2	*Pelobates cultripes*, egg	Spain, Avila, Malpartida	2007	KF718050
SAP0457	*Saprolegnia* sp. 2	*Pelobates cultripes*, egg	Spain, Avila, Malpartida de Corneja	2007	KF718053
SAP0500	*Saprolegnia* sp. 2	Lake	Chile, Saval Lake, Valdivia	2007	KF718055
SAP0624	*Saprolegnia* sp. 2	River	Spain, Avila, Malpartida de Corneja	2007	KF718058
SAP0625	*Saprolegnia* sp. 2	River	Spain, Avila, Malpartida de Corneja	2007	KF718059
SAP0626	*Saprolegnia* sp. 2	River	Spain, Avila, Malpartida de Corneja	2007	KF718060
SAP0653	*Saprolegnia* sp. 2	River	Spain, Oronotz-Mugairi, Navarra	2007	KF718061
SAP0656	*Saprolegnia* sp. 2	River	Spain, Arteta, Sabaiza, Navarra	2007	KF718062
SAP0708	*Saprolegnia* sp. 2	River		2008	KF718063
SAP0709	*Saprolegnia* sp. 2	River		2008	KF718064
SAP0771	*Saprolegnia* sp. 2	River	Ecuador, Jatún Sacha, Napo	2008	KF718065
SAP0776	*Saprolegnia* sp. 2	River	Ecuador, Jatún Sacha, Napo	2008	KF718066
SAP0781	*Saprolegnia* sp. 2	River	Ecuador, Jatún Sacha, Napo	2008	KF718067
SAP0783	*Saprolegnia* sp. 2	River	Ecuador, Jatún Sacha, Napo	2008	KF718068
SAP0792	*Saprolegnia* sp. 2	*Hyloscirtus alytolylax*, egg	Ecuador, Jatún Sacha, Napo	2008	KF718069

SAP0794	*Saprolegnia* sp. 2	River	Spain, Jatún Sacha, Napo	2008	KF718070
SAP0812	*Saprolegnia* sp. 2	*Scinax garbei*, egg	Ecuador, Jatún Sacha, Napo	2009	KF718071
SAP0813	*Saprolegnia* sp. 2	*Scinax garbei*, egg	Ecuador, Jatún Sacha, Napo	2008	KF718072
SAP0819	*Saprolegnia* sp. 2	River	Ecuador, Jatún Sacha, Napo	2008	KF718073
SAP0820	*Saprolegnia* sp. 2	River	Ecuador, Jatún Sacha, Napo	2008	KF718074
SAP0829	*Saprolegnia* sp. 2	River	Ecuador, Jatún Sacha, Napo	2008	KF718075
SAP0900	*Saprolegnia* sp. 2	River	Ecuador, Wusiu, Morona Santiago	2009	KF718076
SAP0933	*Saprolegnia* sp. 2	River	Ecuador, Wusiu, Morona Santiago	2009	KF718077
SAP0955	*Saprolegnia* sp. 2	River	Ecuador, Wusiu, Morona Santiago	2009	KF718078
SAP0971	*Saprolegnia* sp. 2	River	Ecuador, Wusiu, Morona Santiago	2009	KF718079
SAP0987	*Saprolegnia* sp. 2	River	Ecuador, Wusiu, Morona Santiago	2009	KF718080
SAP0988	*Saprolegnia* sp. 2	River	Ecuador, Wusiu, Morona Santiago	2009	KF718081
SAP0992	*Saprolegnia* sp. 2	River	Ecuador, Wusiu, Morona Santiago	2009	KF718082
SAP1006	*Saprolegnia* sp. 2	River	Ecuador, Wusiu, Morona Santiago	2009	KF718083
SAP1008	*Saprolegnia* sp. 2	River	Ecuador, Wusiu, Morona Santiago	2009	KF718084
SAP1028	*Saprolegnia* sp. 2	River	Ecuador, Wusiu, Morona Santiago	2009	KF718085
SAP1101	*Saprolegnia* sp. 2	Lake	Spain, Sierra Nevada, Granada	2010	KF718088
SAP1103	*Saprolegnia* sp. 2	Lake	Spain, Sierra Nevada, Granada	2010	KF718090
SAP1104	*Saprolegnia* sp. 2	Lake	Spain, Sierra Nevada, Granada	2010	KF718091
SAP1298	*Saprolegnia* sp. 2	River	Ecuador, Morona Santiago	2012	KF718093
SAP1302	*Saprolegnia* sp. 2	River	Ecuador, Morona Santiago	2012	KF718094
SAP1303	*Saprolegnia* sp. 2	River	Ecuador, Morona Santiago	2012	KF718095
SAP1304	*Saprolegnia* sp. 2	River	Ecuador, Morona Santiago	2012	KF718096
SAP1311	*Saprolegnia* sp. 2	Lake	Ecuador, Morona Santiago	2012	KF718100
SAP1312	*Saprolegnia* sp. 2	Lake	Ecuador, Morona Santiago	2012	KF718101
SAP1315	*Saprolegnia* sp. 2	Lake	Ecuador, Morona Santiago	2012	KF718102
SAP1316	*Saprolegnia* sp. 2	River	Ecuador, Morona Santiago	2012	KF718103
SAP1323	*Saprolegnia* sp. 2	River	Ecuador, Morona Santiago	2012	KF718104
SAP1326	*Saprolegnia* sp. 2	River	Ecuador, Morona Santiago	2012	KF718105
SAP1330	*Saprolegnia* sp. 2	River	Ecuador, Morona Santiago	2012	KF718106
SAP1370	*Saprolegnia* sp. 2	River	Ecuador, Morona Santiago	2012	KF718107
SAP1436	*Saprolegnia* sp. 2	River	Ecuador, Morona Santiago	2012	KF718110

SAP1439	*Saprolegnia* sp. 2	Lake	Ecuador, Morona Santiago	2012	KF718111
SAP1440	*Saprolegnia* sp. 2	River	Ecuador, Morona Santiago	2012	KF718112
SAP1441	*Saprolegnia* sp. 2	River	Ecuador, Morona Santiago	2012	KF718113
SAP1449	*Saprolegnia* sp. 2	River	Ecuador, Morona Santiago	2012	KF718114
SAP1456	*Saprolegnia* sp. 2	River	Ecuador, Morona Santiago	2012	KF718115
SAP1459	*Saprolegnia* sp. 2	River	Ecuador, Morona Santiago	2012	KF718116
SAP1823	*Saprolegnia* sp. 2	River	Spain, Pirineos, Cataluña	2013	KF718117
SAP1951	*Saprolegnia* sp. 2	River	Spain, Pirineos, Cataluña	2013	KF718119
SAP1975	*Saprolegnia* sp. 2	River	Spain, Pirineos, Cataluña	2012	KF718120
SAP9159	*Saprolegnia* sp. 2	*Oncorhynchus mykiss*, Alevin	Chile, Puerto Montt	2012	KM095758
SAP9160	*Saprolegnia* sp. 2	*Salmo salar*, egg	Chile, Melipeuco	2012	KM095759
SAP9161	*Saprolegnia* sp. 2	*Salmo salar*, Alevin	Chile, Melipeuco	2012	KM095760
SAP9158	*Saprolegnia* sp. 2	*Oncorhynchus mykiss*, egg	Chile, Puerto Montt	2012	KM095761
SAP9162	*Saprolegnia* sp. 2	*Salmo salar*, egg	Chile, Puerto Montt	2012	KM095762
SAP9163	*Saprolegnia* sp. 2	*Salmo salar*, Alevin	Chile, Puerto Montt	2012	KM095763

[1] RJB: Real Jardín Botánico Culture Collection – Madrid, Spain
